# Clinical Usefulness of Immunohistochemical Staining of p57^kip2^ for the Differential Diagnosis of Complete Mole

**DOI:** 10.1155/2015/905648

**Published:** 2015-06-16

**Authors:** Shigeru Sasaki, Yasushi Sasaki, Toshiaki Kunimura, Akihiko Sekizawa, Yoshihiro Kojima, Koichi Iino

**Affiliations:** ^1^Department of Obstetrics and Gynecology, Iino Hospital, 4-3-2 Fuda, Chōfu, Tokyo 182-0024, Japan; ^2^Department of Obstetrics and Gynecology, Showa University Northern Yokohama Hospital and Showa University School of Medicine, 35-1 Chigasaki Chuo, Tsuduki-Ku, Yokohama, Kanagawa 224-8503, Japan; ^3^Department of Pathology, Showa University School of Medicine and Showa University Northern Yokohama Hospital, 35-1 Chigasaki Chuo, Tsuduki-Ku, Yokohama, Kanagawa 224-8503, Japan; ^4^Maternity Clinic Kojima, 4-1-27 Asahi-Cho, Akishima, Tokyo 196-0025, Japan

## Abstract

*Objective*. Can polymer-based immunohistochemical staining of p57^kip2^ replace DNA analysis as an inexpensive means of differentiating complete mole from partial mole or hydropic abortion? *Methods and Materials*. Original paraffin-embedded tissue blocks from 14 equivocal cases were turned over to our laboratory and examined by immunohistochemical staining of p57^kip2^. *Results*. Four of the 14 cases showed clearly negative nuclear staining in cytotrophoblasts and villous stromal cells: these results were fully concordant with the control staining. The remaining 10 cases showed apparently positive staining in cytotrophoblasts and villous stromal cells. Without DNA analysis we are able to clearly differentiate the 4 cases of complete mole among the 14 equivocal cases. During follow-up, secondary low-risk gestational trophoblastic neoplasia (GTN) developed in 1 of the 4 cases of complete mole: the GTN was treated by single-agent chemotherapy. No subsequent changes were observed during follow-up in the other cases. *Conclusion*. Polymer-based immunohistochemical staining of p57^kip2^ (paternally imprinted gene, expressed from maternal allele) is a very effective method that can be used to differentiate androgenetic complete mole from partial mole and hydropic abortion. We might be able to avoid the cost of DNA analysis.

## 1. Introduction

Today, widespread use of ultrasonography and measurement of serum human chorionic gonadotropin (hCG) can be used to detect blighted ovum in the very early stage of pregnancy. Typical classic hydatidiform mole is now rarely seen. However, we, including pathologists, often face equivocal cases of complete mole versus partial mole that are difficult to diagnose histologically. In such cases, pathologists always notify us that complete mole cannot be ruled out and that strict clinical follow-up should be necessary. Usually, we proceed to DNA polymorphism analysis to obtain an accurate diagnosis in such cases. This requires both the patient's consent and extra expenditures.

We recently read the report that p57^kip2^ gene, which encodes the cyclin-dependent kinase inhibitor (CDKI) p57^kip2^, was located on chromosome 11 p15.5 and that this gene is paternally imprinted but expressed from the maternal allele. In the androgenetic complete mole, this gene is underexpressed or not expressed at all as discussed by Saxena et al. [1].

Several reports [2–7] have been published on the efficacy of immunohistochemical staining of this gene product for differentiation of complete mole, although there have been some exceptions. For many years, we have performed this same examination confirmed by DNA analysis in our laboratory. However, we obtained several false-positive results by immunohistochemical staining of p57^kip2^. We noticed that the false-positive immunoreaction was induced by endogenous biotin when we applied the standard streptavidin-biotin method that was used in the reported studies.

The polymer-based method is now gaining traction as an improved method in immunohistochemical staining method. With this method, a secondary antibody conjugated with a polymer is used. This polymer method has 10 to 100 times the sensitivity of the standard method, and there is almost no false staining of the target cells. Before starting this study, we used the polymer method in 10 cases each of androgenetic complete mole, partial mole, and biparental spontaneous abortion. These cases have been diagnosed by DNA analysis in our laboratory.

We confirmed completely negative immunohistochemical staining of p57^kip2^ by this polymer method in cytotrophoblasts and villous stromal cells of the complete moles. Further, there was no false negative staining in the 10 cases of partial moles or the 10 cases of abortion, respectively, in this preliminary study.

We report herein the results obtained by polymer-based immunohistochemical staining of p57^kip2^ in 14 equivocal cases.

## 2. Objective

Can polymer-based immunohistochemical staining of p57^kip2^ replace DNA analysis as an inexpensive means of differentiating complete mole from partial mole or hydropic abortion?

## 3. Materials and Methods

### 3.1. Materials

We investigated 14 cases considered equivocal after evacuation by local doctors. All were local cases referred to us in 2012. All cases were initially diagnosed by pathologists working at commercial clinical laboratories. This is because local doctors ask first for pathological examination by commercial clinical laboratories as a matter of routine management. After equivocal results were returned to these doctors, the specimens were sent to us for further evaluation. Original paraffin-embedded tissue blocks from these 14 cases were collected by our laboratory for this project. These sections were made for each case stained with hematoxylin-eosin and then reexamined independently by our three pathologists and reclassified as difficult equivocal cases. Under our pathologists' review, 5 cases were considered as either hydropic abortion or partial mole, and 9 cases were considered partial or complete mole (Table 1). Informed consent was obtained from all patients, and the 14 cases were examined by polymer-based immunohistochemical staining of p57^kip2^.

### 3.2. Methods

The polymer-based method of immunohistochemical staining is now well known to have 10 to 100 times the sensitivity of the standard streptavidin-biotin method. The immunohistochemical staining was carried out by heat-induced antigen retrieval followed by the polymer method. Duplicated 4 *µ*m thick sections from the formalin-fixed, paraffin-embedded blocks were obtained in each case. Sections were deparaffinized in xylene and alcohol, washed, and rehydrated in distilled water.

After endogenous peroxidase activity was quenched with 3% hydrogen peroxidase solution, antigen retrieval was performed. The sections were immersed in 0.01 M citrate buffer (pH 7.0) with 0.1% Tween-20, kept for 40 minutes at 98°C. The sections were allowed to cool for 20 minutes spontaneously. Next, sections were immersed in 1 mM EDTA (pH 9.0), for 40 minutes at 98°C, and again allowed to cool. Next the sections were immersed again in 1 mg/mL protease XXIV (Sigma-Aldrich. St. Louis, MO, USA) in PBS for 60 minutes at room temperature and then washed in water and PBS. To block nonspecific reactions, these sections were immersed with 5% gout serum for 20 minutes at room temperature. Mouse monoclonal antibody for human p57^kip2^ protein, the primary antibody, was applied to samples for overnight incubation at 4°C (Novocastra Liquid Mouse Monoclonal Antibody for human p57 protein (Product code: NCL-L-p57: Leica Biosystems Newcastle Ltd, Newcastle, UK)). Peroxidase-labeled secondary antibody for anti-mouse immunoglobulin conjugated with amino acid polymer (Nichirei Co, Ltd., Tokyo, Japan) was applied for 60 minutes at room temperature. Sections were then washed three times for 5 minutes each with PBS. The sections were incubated with diaminobenzidine as a chromogen for 10 minutes, then washed in water, and nuclear-counterstained with hematoxylin. Staining patterns on the tissue sections were examined microscopically and compared to those of control sections. The control sections were prepared from the DNA-established androgenetic complete moles, partial moles (triploidy), and abortions of biparental origin and prepared in the same manner as the cases' sections.

**Figure 1:**
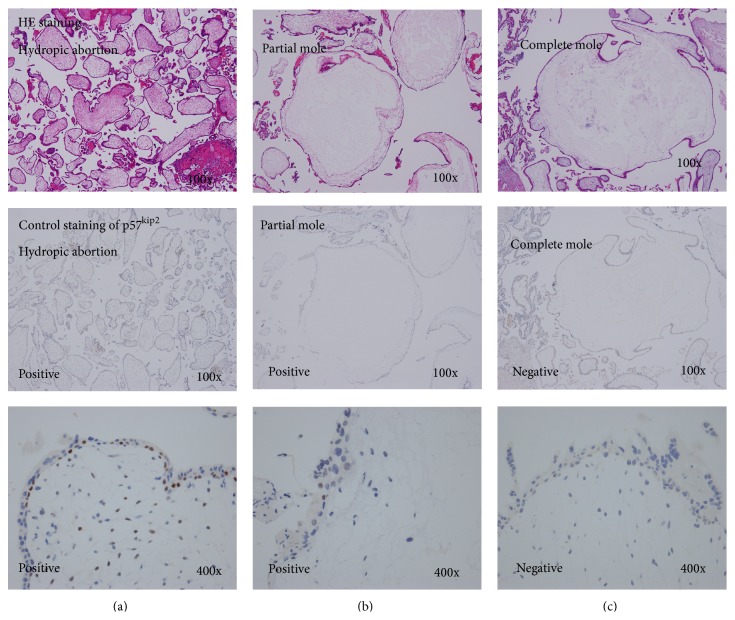
Those figures are the control staining in hydropic abortion (left), partial mole (middle), and complete mole (right). Upper panels show hematoxylin-eosin staining, and middle and lower panels show immunohistochemical staining for p57^kip2^ by the polymer-based method of control hydropic abortion (left), partial mole (middle), and complete mole (right). Magnification are 100x and 400x by microscope, respectively. In cytotrophoblasts and villous stromal cells, strong positive immunoreactive staining for p57^kip2^ is seen in the hydropic abortion (left) and partial mole (middle): staining is absent in the complete mole (right). The genetic origins of the control partial and complete mole were established by DNA polymorphism analysis in our laboratory.

Reactivity was judged positive only when distinct nuclear staining of cytotrophoblasts and villous stromal cells was identified. There was no faint nuclear staining observed by polymer-based method through this experiment. Control study showed clearly negative staining of complete moles and positive in partial moles and abortions in cytotrophoblasts and villous stromal cells, respectively. Decidual stromal cells were positive for p57^kip2^ in all cases and provided a reliable internal control (Figure 1). Syncytiotrophoblasts in complete moles, partial moles, and abortions always stained negatively (Figure 1).

## 4. Results

Duplicate immunohistochemical staining by the polymer method was done for each of the 14 equivocal cases, and the staining patterns were compared with those of the control cases confirmed genetically in our laboratory. Four cases (Cases 2, 5, 6, and 9 indicated by asterisks in Table 1) showed a clearly negative immunoreaction for p57^kip2^. The others stained positively.

Thus, we were able to differentiate these 4 cases as complete moles among the 9 equivocal cases of partial or complete mole. Cases 3, 4, 10, 11, and 12 in Table 1 were considered partial moles.

This staining did not differentiate partial moles from hydropic abortions.

The other cases (Cases 1, 7, 8, 13, and 14 in Table 1) remained equivocal cases of partial mole, or hydropic abortion.

These 4 cases of complete moles as well as the other cases were followed to 24 weeks by weekly serum hCG measurement. Of the 4 cases of complete mole, one (Case 5 in Table 1) developed into a secondary low risk gestational trophoblastic neoplasia (GTN) and was treated with single-agent chemotherapy.

No subsequent changes were observed during follow-up in the other cases.

## 5. Discussion

As a preliminary study, we performed standard streptavidin-biotin immunohistochemical staining of p57^kip2^ in our DNA-established complete mole and hydropic abortion cases to know how effective the reported method is for differentiation of complete moles from hydropic abortion [5, 6]. In several established moles, however, we observed false positive staining. In reading the previous papers carefully, we learned that the investigators also encountered a small percentage of false positive staining.

With the standard streptavidin-biotin method, endogenous biotin has a positive effect on the staining pattern. So, we then used 3% hydrogen peroxidase solution to quench the endogenous biotin activity. This was 10 times the concentration reported by Jun et al. [6], but we still encountered false positive staining in several established complete moles. Subsequently, we learned that the polymer method of immunohistochemical staining, in which a secondary antibody conjugated with a polymer is used, is much more sensitive (10 to 100 times) than the standard streptavidin-biotin method.

The polymer method is easy and more sensitive, and it is not affected by endogenous biotin.

The secondary antibody conjugated with a polymer can be easily obtained commercially.

The polymer-based method is now described in textbooks as an improved method.

We applied the polymer method to our DNA analysis-established androgenetic complete moles and confirmed that the polymer methods do not produce false-positive or false-negative staining.

We found the method to be a reliable tool that can be used to differentiate complete mole in equivocal cases without the need for DNA analysis of each specimen.

However, there is 1 report of a definitive androgenetic complete mole that stained positively for p57^kip2^ [3].

Of course, we must be vigilant, and we must realize that immunoreaction is not always absolute.

DNA analysis should be done, whenever a case remains questionable. However, there is no doubt that the polymer method is sensitive and effective.

We believe this method to be a very useful tool for differentiation of complete mole, when the results of other tests are equivocal. We would like to recommend that the polymer method of immunohistochemistry be applied first as a routine examination in any equivocal cases, especially for doctors who work in developing countries, where DNA analysis is far too expensive or even unfeasible. We may be able to avoid the cost of DNA analysis.

Kihara et al. published the first report of perfect concordance between negative p57^kip2^ immunoreactivity and molar tissue of androgenetic origin [8]. They used a polymer system produced by DakoCytomation (Glostrup, Denmark). Our study independently supports their findings.

## 6. Conclusion

Polymer-based immunohistochemical staining of p57^kip2^ (paternally imprinted gene, expressed from maternal allele) is a very effective method that can be used to differentiate androgenetic complete mole from partial mole and hydropic abortion. We might be able to avoid the cost of DNA analysis.

## Figures and Tables

**Table 1:** Fourteen equivocal cases subjected to polymer-based p57^kip2^ immunohistochemistry for differentiation between complete and partial mole or hydropic abortion.

Case/patient	Age (yr)	G-P-A	Clinical Dx^*^	hCG mIU/mL before evacuation	Histopathologic Dx	p57^kip2^ staining	Final Dx
1	40	1-0-1	7 weeks	6700	Hydropic/partial	+	Hydropic/partial
2^**^	30	3-3-0	7 weeks	4780	Partial/complete	−	Complete mole

3	40	2-2-0	7 weeks	49100	Partial/complete	+	Partial
4	27	3-2-1	8 weeks	83100	Partial/complete	+	Partial
5^∗∗†^	48	4-2-2	6 weeks	6590	partial/complete	−	Complete mole

6^**^	30	3-1-2	7 weeks	28500	Partial/complete	−	Complete mole

7	27	1-1-0	8 weeks	91700	Hydropic/partial	+	Hydropic/partial
8	44	3-1-2	7 weeks	4670	Hydropic/partial	+	Hydropic/partial
9^**^	27	1-1-0	6 weeks	31200	Partial/complete	−	Complete mole

10	34	2-1-1	7 weeks	7800	Partial/complete	+	Partial
11	34	2-1-1	8 weeks	4400	Partial/complete	+	Partial
12	33	0-0-0	5 weeks	4600	Partial/complete	+	Partial
13	32	1-1-0	6 weeks	6800	Hydropic/partial	+	Hydropic/partial
14	36	3-2-1	6 weeks	5200	Hydropic/partial	+	Hydropic/partial

G-P-A, gravida/para/abortus; hCG, human chorionic gonadotropin; dilation and curettage; Dx, diagnosis.

^*^All diagnosed clinically as blighted ovum; ^**^clearly differentiated as complete hydatidiform mole by polymer-based immunohistochemistry for p57^kip2^; ^†^hCG elevated to 8740, persistent trophoblastic disease, treated by single-agent chemotherapy.

S. SASAKI 2012.
